# Driving in Home Treatment Teams: Are We Talking About It Enough? An Audit Covering Kingston and Richmond Boroughs in South West London and St George's NHS Trust

**DOI:** 10.1192/bjo.2024.575

**Published:** 2024-08-01

**Authors:** Soracha Healy, Radhika Lakhani, Zulkarnain Ahmad, Sasha Francis

**Affiliations:** South West London and St George's NHS Trust, London, United Kingdom

## Abstract

**Aims:**

Legality of driving and serious mental illness is often poorly understood by service users and staff. The risk of rare but serious consequences indicate the value in including driving risk in initial assessments. The Driving and Vehicle Licensing Authority (DVLA) advises not to drive and notify them of changes in condition or concerns around an individual's ability to drive. Crisis periods can represent changes in condition for individuals with chronic mental health conditions including psychotic disorders, manic episodes, severe anxiety and depression, and personality disorders. It therefore is pertinent for home treatment team (HTT) clinicians to consider driving safety, in patients requiring crisis intervention. The aim of our audit was to identify what proportion of patients on the Kingston and Richmond HTT caseloads are asked about driving and implement changes to facilitate discussion thereby improving safety.

**Methods:**

Retrospective data was collected from Rio clinical record software from the entire Richmond HTT and Kingston HTT caseloads at baseline, two and four months post-intervention. Clinical records were reviewed to establish if driving was being discussed. Data was inputted anonymously into Excel and simple statistical analyses conducted.

Inclusion criteria were patients on the Richmond Kingston HTT caseloads on the date of data extraction for cycles 1, 2 and 3. Patients were excluded who had not yet had their initial assessment.

Following initial data collection we joined stakeholders at Trust-Wide HTT Governance meeting covering five boroughs and presented findings. We agreed changes to implement including incorporating a driving prompt in the initial assessment proforma and providing a DVLA leaflet in the welcome pack.

**Results:**

From baseline data of combined caseloads, 17.7% of patients had documented evidence of driving discussion. At two months, re-audit showed that 33.3% of patients were asked about driving. With consideration of delays in change implementation with large teams and shift work, a third data collection cycle was completed 4 months post intervention. This showed that 56.0% of patients were asked about driving.

**Conclusion:**

The changes implemented have been effective in sustaining increased awareness on this important topic and facilitating discussion with patients. There is potential to increase awareness further by expanding this as a trust-wide, regional or national initiative whilst enhancing stakeholder engagement.

